# Strong mucosal immune responses in SIV infected macaques contribute to viral control and preserved CD4+ T-cell levels in blood and mucosal tissues

**DOI:** 10.1186/1742-4690-8-24

**Published:** 2011-04-11

**Authors:** Tina Schultheiss, Reiner Schulte, Ulrike Sauermann, Wiebke Ibing, Christiane Stahl-Hennig

**Affiliations:** 1 Unit of Infection Models, German Primate Center, Leibniz Institute for Primate Research, Kellnerweg 4, 37077, Goettingen, Germany; 2 Cancer Research UK Cambridge Research Institute, Li Ka Shing Centre, Robinson Way, Cambridge CB2 0RE, UK

## Abstract

**Background:**

Since there is still no protective HIV vaccine available, better insights into immune mechanism of persons effectively controlling HIV replication in the absence of any therapy should contribute to improve further vaccine designs. However, little is known about the mucosal immune response of this small unique group of patients. Using the SIV-macaque-model for AIDS, we had the rare opportunity to analyze 14 SIV-infected rhesus macaques durably controlling viral replication (controllers). We investigated the virological and immunological profile of blood and three different mucosal tissues and compared their data to those of uninfected and animals progressing to AIDS-like disease (progressors).

**Results:**

Lymphocytes from blood, bronchoalveolar lavage (BAL), and duodenal and colonic biopsies were phenotypically characterized by polychromatic flow cytometry. In controllers, we observed higher levels of CD4+, CD4+CCR5+ and Gag-specific CD8+ T-cells as well as lower immune activation in blood and all mucosal sites compared to progressors. However, we could also demonstrate that immunological changes are distinct between these three mucosal sites.

Intracellular cytokine staining demonstrated a significantly higher systemic and mucosal CD8+ Gag-specific cellular immune response in controllers than in progressors. Most remarkable was the polyfunctional cytokine profile of CD8+ lymphocytes in BAL of controllers, which significantly dominated over their blood response. The overall suppression of viral replication in the controllers was confirmed by almost no detectable viral RNA in blood and all mucosal tissues investigated.

**Conclusion:**

A strong and complex virus-specific CD8+ T-cell response in blood and especially in mucosal tissue of SIV-infected macaques was associated with low immune activation and an efficient suppression of viral replication. This likely afforded a repopulation of CD4+ T-cells in different mucosal compartments to almost normal levels. We conclude, that a robust SIV-specific mucosal immune response seems to be essential for establishing and maintaining the controller status and consequently for long-term survival.

## Background

Over 33 million people are infected with HIV worldwide. Since there is currently no protective vaccine available, the understanding of viral-host interactions and immune responses in the small number of HIV-infected individuals demonstrating robust control of systemic HIV replication over long periods of time, in the absence of any therapy, should advance the design of new vaccines.

The majority of studies are focused on systemic immune responses which correlate with low viral loads [1-3], even though the mucosal immune system plays not only a central role in HIV transmission [4,5], but also in the pathogenesis of AIDS [6-8]. The dramatic loss of CD4+ T-cells in all mucosal tissue is a hallmark of early HIV infection [9-12], which subsequently leads to several local opportunistic infections and contributes to AIDS [13-15]. In particular, high viral replication in the gut is accompanied by gut atrophy [16], malabsorption [17], chronic diarrhea and weight loss [6,18].

The experimental infection of rhesus macaques (RM) with simian immunodeficiency virus (SIV) has been intensively utilized as a model to investigate the pathogenesis of human HIV infection. Approximately 5% of RM of Indian origin are able to control SIV replication [19] which is similar to the rate reported in HIV-infected humans [20,21]. Therefore, larger cohorts of such animals have rarely been studied, and in particular their viral kinetics and virus-specific immune responses at different mucosal sites have not yet been comprehensively investigated.

In this study, we had the unique opportunity to investigate 14 SIV-infected RM of Indian origin, which have been effectively suppressing systemic viral load for several years (controllers) in comparison to uninfected animals and SIV-infected RM with high viral loads and a more rapid disease progression (progressors). We aimed to investigate if and how the mucosal immune system contributes to the control of viral replication, and we performed detailed analyses of three distinct mucosal sites *ex vivo*.

Intestinal biopsies from duodenum and colon were obtained, and lung cells were collected via bronchoalveolar lavage (BAL) in parallel. Paired blood samples and mucosal lymphocytes were characterized by analyzing their phenotypic composition and SIV-specific T-cell function. In addition, the viral load was determined in blood and all mucosal sites by quantifying viral RNA and proviral DNA load.

## Results

### Baseline characteristics of SIV infected RM

This study included 30 SIV-infected rhesus monkeys of Indian origin infected with SIVmac239 or SIVmac251. All animals are listed in Table 1 which indicates the period of investigation and assays performed, together with their respective mean viral load in plasma during that time. 12 of the 14 controllers carried MHC alleles associated with slow disease progression. 10 RM (70%) carried Mamu-A1*001 and six RM had Mamu-B*017 (43%) (Additional file 1). Four of the latter carried also Mamu-A1*001.

**Table 1:** Animals and assays performed

Animal	SIVmac strain	Route of infection	Period of investigation^1 ^	Average plasma viral RNA load	FACS	CM9	ELISpot	ELISA	ICS	Viral RNA load			Proviral load			
										**D**	**C**	**B**	**D**	**C**	**B**	**P**
Controllers																
2139*	239	tonsillar	63-245	1.1 × 10^2^	X	X	X	X	X	X	X	X	X	X	X	X
2151*	239	tonsillar	63-245	8.4 × 10^1^	X	X	X	X	X	X	X	X	X	X	X	X
2153*	239	tonsillar	64-245	1.2 × 10^2^	X	X	X	X	X	X	X	X	X	X	X	X
2155*	239	tonsillar	63-245	1.1 × 10^2^	X	X	X	X	X	X	X	X	X	X	X	X
2172	239	tonsillar	68-245	2.5 × 10^2^	X	X	X	X	X	X	X	X	X	X	X	X
2191*	239	tonsillar	71-146	3.8 × 10^3^	X	X		X	X							
8644*	251	tonsillar	444-550	5.5 × 10^2^	X			X	X	X	X	X	X	X	X	X
9045*	239	i.v.	490-507	1.8 × 10^4^	X			X	X							
9794	239	tonsillar	209-315	1.2 × 10^3^	X			X	X	X	X	X	X	X	X	X
12533*	239	tonsillar	68-116	2.0 × 10^3^	X	X	X	X								
12535	239	tonsillar	71-153	1.3 × 10^2^	X		X	X	X	X	X	X				
12536*^,2^	239	tonsillar	67-157	4.3 × 10^2^	X	X	X	X	X	X	X	X				
12671*	239	tonsillar	68-241	9.9 × 10^1^	X	X	X	X	X	X	X	X	X	X	X	X
12672	239	tonsillar	71-241	1.7 × 10^2^	X		X	X	X	X	X	X	X	X	X	X
Progressors																
2118*	239	tonsillar	99-177	1.7 × 10^5^	X	X		X	X			X			X	X
2141	239	tonsillar	104-120	6.5 × 10^4^	X		X									
2188*	239	tonsillar	107-124	3.1 × 10^5^	X	X	X									
12537	239	tonsillar	102-107	7.5 × 10^5^	X											
2168	239	tonsillar	112-116	1.1 × 10^5^	X		X									
10425	239	tonsillar	113-116	3.7 × 10^4^	X		X									
12539	239	tonsillar	116-117	3.2 × 10^5^	X		X									
2192	251	i.v.	68-92	9.3 × 10^4^	X		X	X		X	X					
12531	239	tonsillar	128-146	4.8 × 10^4^	X		X	X	X	X	X					
12538*	251	i.v.	85-115	2.9 × 10^5^	X			X	X	X	X				X	X
11139*	251	i.v.	69-121	1.1 × 10^5^	X	X	X	X	X	X	X	X			X	X
13251*	251	i.v.	69-115	2.0 × 10^5^	X	X	X	X	X	X	X	X			X	X
13258	251	i.v.	96-105	8.2 × 10^5^			X	X	X	X	X	X				
13250*	251	i.v.	105-115	1.1 × 10^5^			X	X	X			X			X	X
13257	251	i.v.	105-115	2.8 × 10^5^			X	X	X			X				
13260	251	i.v.	92-101	1.2 × 10^5^			X			X	X	X				

*, animals expressing the MHC class I allele MamuA1*001

^1^, weeks post infection

^2^, this animal had an increasing viral load after week 160 post infection, but was separately analyzed until week 250 post infection

FACS, flow cytometric phenotype staining; CM9, Gag-CM9 tetramer staining; D, duodenum; C, colon; B, BAL; P, PBMC; ICS, intracellular cytokine staining

**Figure 1:**
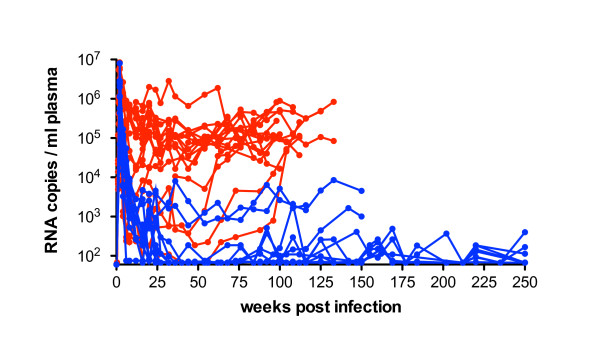
**SIV viral RNA load in plasma of controllers and progressors**. Viral RNA copies per ml plasma are shown during infection with SIVmac239 or SIVmac251 until necropsy or exclusion from study. Controllers are depicted in blue, progressors in red. Mean peak viremia was similar in both groups, but from week 8 p.i. onward controllers exhibited a significantly lower viral load than progressors (*P *< 0.05 Mann-Whitney's *U*-test). The detection limit for this assay was 75 viral RNA copies per ml plasma. Viral loads of the long term infected monkeys 9045, 8644, 9794 are not shown.

The controllers reduced viral replication soon after peak viremia and were defined by maintaining a mean viral load of less than 5 × 10^3 ^RNA copies per ml plasma (Figure 1) except for one animal (9045). Although this monkey had a viral load above 1 × 10^4 ^copies/ml plasma, it was included in the controller group due to its extremely long survival for more than 10 years. The progressors were defined as having viral loads above 10^4 ^viral RNA copies/ml plasma during the period of investigation (Table 1). However, it should be noted that they represent slow progressors as their survival time.

### Higher levels of CD4+ T-cells in blood, BAL and gut of controllers compared to progressors

The loss of CD4+ T-cells in blood during HIV/SIV infection is generally modest, whereas mucosal tissues represent the major site of viral replication. Since most of the mucosal CD4+ T-cells are activated memory cells expressing the viral coreceptor CCR5 [22-24], viral replication leads to a massive and almost complete depletion of CD4+ T-cells in all stages of infection [12,22,25,26].

Flow cytometric analysis was performed to investigate the proportion of CD4+ and CD4+CCR5+ T-cells in blood, BAL, duodenum and colon of SIV-infected controllers and progressors in comparison to uninfected animals.

**Figure 2:**
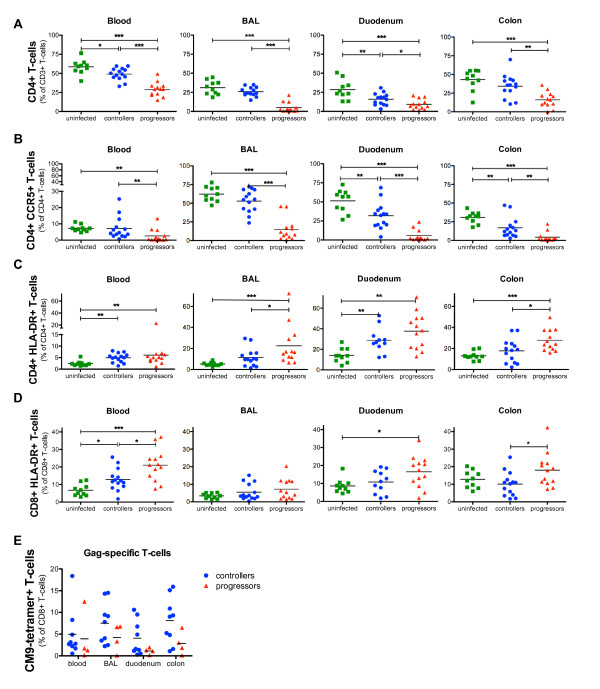
**T-cell analyses in blood, BAL, duodenum and colon of controllers, progressors and uninfected RM**. Flow cytometric analyses of (A) CD4+ T-cells, (B) CD4+CCR5+ T-cells, (C) CD4+HLA-DR+ T-cells, (D) CD8+ HLA-DR+ T-cells in blood, BAL, duodenum and colon of controllers, progressors and uninfected animals. (E) SIV-Gag specific T-cells were detected with CM9-tetramers in blood, BAL, duodenum and colon of Mamu-A1*001 controllers (blue) and progressors (red). Horizontal lines represent the mean of each group and *P*-values were calculated with the Mann-Whitney's *U*-test (*P < 0.05, **P < 0.01 and ***P < 0.001).

The fraction of CD4+ T-lymphocytes in blood and duodenum was significantly reduced in controllers compared to uninfected RM (49% vs 58% *P <*0.05; 16% vs 29% *P <*0.01), but interestingly controllers maintained almost normal CD4+ T-cell levels in BAL (26%) and colon (34%) (Figure 2A).

Analyzing CD4+CCR5+ T-cells in blood and BAL of controllers revealed no significant difference compared to uninfected monkeys whereas a reduced proportion of this T-cell subset was observed in both intestinal sites (Figure 2B). In contrast, progressors displayed in blood and all mucosal sites significantly lower levels of CD4+ and CD4+CCR5+ T-cells than controllers and uninfected animals (Figure 2A, B).

The analysis of all SIV-infected animals revealed a highly significant inverse correlation between the viral RNA load in plasma and the CD4+ T-cells in blood (*P *< 0.0001; r = -0.786), BAL (*P *< 0.0001; r = -0.814), duodenum (*P *= 0.008; r = -0.497) and colon (*P *< 0.0001; r = -0.685) as well as for the proportion of CD4+CCR5+ T-cells in blood (*P *= 0.0003; r = - 0.647), BAL (*P *< 0.0001; r = - 0.817), duodenum (*P *< 0.0001; r = - 0.742) and colon (*P *= 0.0003; r = - 0.674).

### Low immune activation in blood and mucosal tissues of controllers

Chronic activation of T-lymphocytes is known to contribute to viral replication and disease progression [27,28]. Therefore, the activation profile of blood and mucosal CD4+ and CD8+ T-cells was analyzed by the expression of the activation marker HLA-DR.

Blood and duodenal CD4+ T-cells of SIV-infected controllers expressed significantly higher levels of HLA-DR in comparison to uninfected RM (blood 4.9% vs 2.4%, *P *< 0.01; duodenum 28% vs 14%, *P *< 0.01), but no significant activation was observed in BAL or colonic samples of these animals (Figure 2C). In contrast, progressors had significantly higher levels of activated CD4+ T-cells in all compartments compared to uninfected RM.

The level of CD8+HLA-DR+ T-cells in blood from controllers was significantly higher than in uninfected animals (6% vs 13%, *P *< 0.05), but in all mucosal sites this T-cell subset did not differ from uninfected macaques (Figure 2D). A significantly higher activation of CD8+ lymphocytes in gut and blood from progressors was observed compared to uninfected RM and controllers, respectively. We observed a significant correlation between the viral RNA copies/ml plasma and the HLA-DR+CD4+ BAL T-cells (*P *= 0.034; r = 0.408) and HLA-DR+CD8+ colonic T-cells (*P *= 0.007; r = -0.507).

### High frequencies of SIV-Gag-specific T-cells in blood and mucosal tissues of controllers

The MHC class I allele Mamu-A1*001 in RM of Indian origin is associated with a lower viral set point and longer survival during SIV infection [29]. Mamu-A1*001 positive RM develop virus-specific cytotoxic CD8+ T-lymphocytes directed against the immune dominant SIV-Gag-CM9-peptide (Gag_181-189_, CTPYDINQM) which can be detected by tetramer staining [30]. We investigated these SIV-Gag-specific T-cells in blood, BAL, duodenum and colon of 13 Mamu-A1*001 positive RM encompassing nine controllers and four progressors.

Overall, in controllers the mean values of CM9-Gag-specific T-cells were slightly higher with 7.5% and 8% (of CD8+ T-cells) in BAL and colon, respectively, compared to blood and duodenum where the mean levels ranged between 4% and 5% (Figure 2E). In contrast, the proportion of Gag-specific cells was lower in all compartments of progressors in comparison to controllers, but these differences did not reach statistical significance probably due to the low number of Mamu-A1*001 progressors available for this assay.

### Association between the proportion of CD4+ T-cells, their HLA-DR expression and the proportion of Gag-specific T-cells in blood and mucosal sites of controllers

It is well known that systemic immune activation correlates with the loss of peripheral CD4+ T-cells and disease progression [31,32]. However, when analyzing blood and three mucosal sites of controllers we observed differences in CD4+ T-cell depletion, immune activation and the levels of Gag-specific T-cells between these compartments (Figure 2).

Blood and duodenum of controllers exhibited significantly decreased levels of CD4+ T-cells and a significantly higher expression of HLA-DR on the CD4+ cells compared to uninfected RM, together with rather lower proportions of Gag-specific CD8+ T-cells (than in BAL and colon) (Figure 2A,C,E). In contrast, BAL and colon exhibited higher levels of Gag-specific T-cells (than blood and duodenum) and displayed no significant difference in the proportion of CD4+ and CD4+HLA-DR+ T-cells compared to uninfected animals (Figure 2A,C,E). These facts displayed a relationship between immune activation, virus-specific immune response and CD4+ T-cell numbers for single compartments.

### Long-term analyses revealed stable proportions of CD4+ and Gag-specific T-cells in blood and mucosal sites of controllers

**Figure 3:**
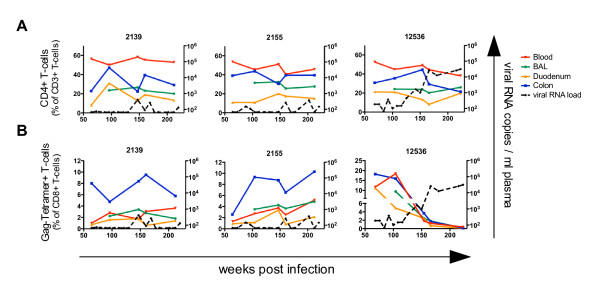
**Long-term analyses of blood and mucosal CD4+ and Gag-specific T-cells in SIV infected RM**. Long-term flow cytometric analyses of (A) CD4+ T-cells and (B) CD8+ CM9-tetramer+ T-cells in blood (red), BAL (green), duodenum (yellow) and colon (blue) of three SIV-infected animals together with plasma viral RNA load (dashed line). Two representative controllers (2139+2155) effectively controlling viral replication (A+B left and middle panels) are shown. One RM (12536) defined as controller until week 150 p.i., was then excluded from the controller group due to its gradually increasing plasma viral load but further investigated until week 220 p.i. (A+B right panels).

Blood and mucosal lymphocytes from 10 (seven of them Mamu-A1*001 positive) controllers were investigated for up to three years. During this period, nine of these animals had continuously low viral loads and permanently high proportions of CD4+ T-cells in blood and all mucosal tissues. In Mamu-A1*001 positive animals we observed also relatively stable levels of Gag-CM9+CD8+ T-cells. The proportions of CD4+ and Gag-specific T-cells of two representative RM (2139+2155) are shown in Figure 3A+B (left and middle panel). In mucosal tissues some variations were observed in the CD4+ and the Gag-CM9+CD8+ T-cell subset, mainly in both gut sites suggesting a local dynamic balance between viral replication and immune response.

In one RM (12536), the viral load slowly increased from 4.5 × 10^2 ^to 3.2 × 10^4 ^viral RNA copies in plasma between weeks 125 to 220 post infection. The increasing viral replication was accompanied by a dramatic loss of Gag-specific T-cells from about 5-20% to 0.1-0.4% (of CD8+ T-cells) in blood and all mucosal sites (Figure 3B, right panel). However, no significant decrease of CD4+ T-cells was observed in blood or mucosal tissues (Figure 3A, right panel).

### Strong humoral and cellular immune response against Gag in controllers

To investigate the breadth of the virus-specific immune response in controllers and progressors, the humoral response in blood against the SIV core protein p27 and the Env protein gp130 was assessed by ELISA, and the cellular one by IFN-γ ELISpot against four different viral peptide pools.

**Figure 4:**
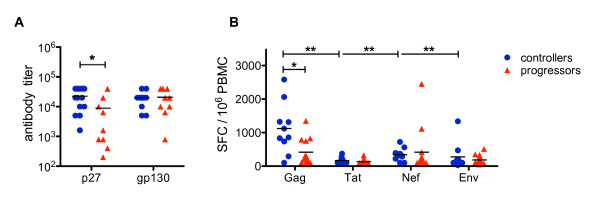
**Systemic virus-specific humoral and cellular immune responses in controllers and progressors**. (A) Antibody titer against the SIV-p27 and SIV-gp130 protein were determined in serum of controllers (blue) and progressors (red) by ELISA. (B) INF-γ secreting lymphocytes as determined by ELISpot assay are shown as the number of spot forming cells (SFC) per 10^6 ^PBMC after stimulation with viral peptide pools SIV-Gag, SIV-Tat, SIV-Nef and SIV-Env in controllers (blue) and progressors (red). Horizontal lines represent the mean of each group and *P*-values were calculated with the Mann-Whitney's *U*-test (*P < 0.05 and **P < 0.01).

Controllers had significantly higher binding antibody titers against p27 compared to progressors, while the titers against gp130 were similar in both animal cohorts (Figure 4A). After stimulation of peripheral blood mononuclear cells (PBMC) with Gag-peptides, the controller group had almost three times the number of IFN-γ secreting cells per 10^6 ^PBMC than progressors (mean 1112 vs 385, *P *= 0.015) (Figure 4B). In contrast, after stimulation with Tat, Nef or Env peptide pools the response was similar in both animal cohorts. Of note, the IFN-γ response of controllers against Gag-peptides dominated significantly over those against all other SIV-peptide pools investigated (*P *< 0.01) (Figure 4B).

### BAL cells from controllers have a higher potential to secrete cytokines upon polyclonal stimulation than those from progressors

T-cells that secrete multiple cytokines upon virus-specific stimulation are associated with the control of viral replication during HIV infection [33-35]. However, beside a virus-specific stimulation we also wanted to compare the general potential of systemic and mucosal T-cells to produce cytokines. We performed ICS with PBMC and BAL cells from uninfected and SIV-infected monkeys detecting the cytokines TNF-α, IFN-γ and IL-2 after polyclonal stimulation with *Staphylococcus *enterotoxin B (SEB).

Boolean gating was applied to determine the proportion of CD45RA- polyfunctional memory T-cells (cells secreting two or all three cytokines). The total response is the percentage of cells responding to SEB and is composed of polyfunctional cells and cells secreting one cytokine only.

**Figure 5:**
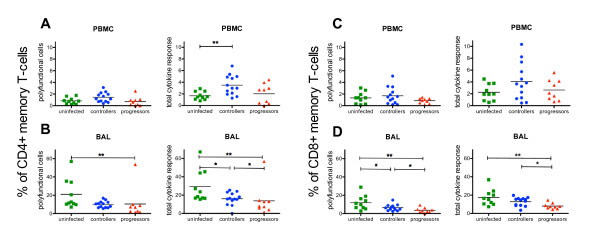
**Cytokine response in PBMC and BAL of controllers, progressors and uninfected animals after polyclonal stimulation**. Percentage of polyfunctional cells and total cytokine secreting cells after SEB stimulation in the CD4+ memory T-cell subset of PBMC (A) and BAL (B) as well as in the CD8+ memory T-cell subset of PBMC (C) and BAL (D) in controllers, progressors and uninfected animals. Polyfunctional cells were defined as expressing two or three cytokines (IFN-γ+ TNF-α+, IFN-γ+ IL-2+, TNF-α+ IL-2+, IFN-γ+ TNF-α+ IL-2+) and the total response comprises polyfunctional cells and cells secreting one cytokine only (single positive cells). Horizontal lines represent the mean of each group and *P*-values were calculated with the Mann-Whitney's *U*-test (*P < 0.05 and **P <  0.01).

After stimulating PBMC from uninfected animals with SEB, we observed about 2% cytokine secreting cells in both CD4+ and CD8+ memory T-cell subsets, whereas half of them were polyfunctional (Figure 5A,C). Compared to PBMC a significantly higher total cytokine response was observed in BAL cells in the CD4+ (29.1% vs 1.7%; *P *< 0.0001) and the CD8+ (17.4% vs 2.25%; *P *< 0.0001) memory T-cell subset including also significantly more polyfunctional CD4+ (20.9% vs 1.33%; *P *< 0.0001) and CD8+ (12% vs 0.8%; *P *< 0.0001) T-cells (Figure 5B,D). These results clearly demonstrate that BAL cells have a higher capability to secrete cytokines compared to PBMC.

Stimulated PBMC of controllers contained significantly higher values of total cytokine secreting CD4+ T-cells compared to uninfected RM (3.5% vs 2.25%; *P *< 0.005), but not higher polyfunctional ones (Figure 5A). Beyond that no further differences in peripheral cytokine secretion were observed between controllers, progressors and uninfected animals (Figure 5A, C).

However, in BAL from the controllers the total level of CD4+ memory cytokine secreting cells, but not the level of polyfunctional cells, was significantly decreased compared to uninfected animals (Figure 5B). BAL CD8+ T-cells of controllers displayed lower proportions of polyfunctional cells, but no difference in the total level of cytokine secreting cells (Figure 5D). The progressors showed significantly lower proportions of polyfunctional and total cytokine secreting CD4+ and CD8+ T-cells compared to uninfected RM and mostly also to controllers (Figure 5B,D).

### Strong polyfunctional virus-specific CD8 T-cell response in BAL of controllers

Based on the dominating systemic Gag-specific IFN-γ ELISpot responses in controllers (Figure 4), the further investigation of cellular immune responses by ICS was focused on Gag. PBMC and BAL cells were stimulated with a Gag-peptide pool and those from Mamu-A1*001 positive animals additionally with the immune dominant CM9-peptide alone.

**Figure 6:**
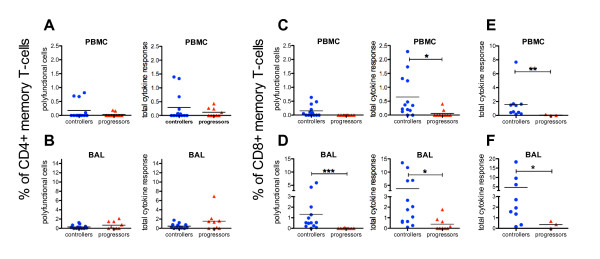
**Virus-specific cytokine response of PBMC and BAL memory T-cells from controllers and progressors**. Percentage of polyfunctional cells and total cytokine secreting cells after SIV-Gag stimulation in the CD4+ memory T-cell subset of PBMC (A) and BAL (B) and in the CD8+ memory T-cell subset of PBMC (C) and BAL (D) in controllers, progressors and uninfected animals. The right panels show the total cytokine response of CD8+ memory T-cells in PBMC (E) and BAL cells (F) of Mamu-A1*001 positive controllers and progressors after stimulation with the CM9-peptide only. For definition of polyfunctional cells and the total response see figure legend 5. Horizontal lines represent the mean of each group and *P*-values were calculated with the Mann-Whitney's *U*-test (*P < 0.05, **P < 0.01 and ***P < 0.001).

The mean values of all CD4+ cytokine secreting cell subsets ranged from 0.12% to 1.52% (of CD4+ memory T-cells) in PBMC and BAL from both SIV-infected animal cohorts. No differences were observed between controllers and progressors in their frequencies of polyfunctional and total cytokine secreting CD4+ memory T-cells in blood and mucosa (Figure 6A,B).

In contrast, striking differences were found in the CD8+ memory T-cell subset. Controllers had 0.65% of CD8+ cytokine secreting cells against Gag in PBMC and 3.7% in BAL being significantly higher compared to 0.06% and 0.38% in progressors (Figure 6C,D, right panels). In addition, 1.3% of the Gag-specific BAL response in controllers was polyfunctional and significantly higher than that in progressors where such a response was almost entirely missing (Figure 6D, left panel). Comparing Gag-specific blood and BAL responses of controllers revealed in BAL, a more than 5-fold higher total CD8+ restricted cytokine secretion (*P *= 0.014) and 8.7-fold higher levels of polyfunctional cells (*P *= 0.004).

For the analyses of Gag-CM9-specific CD8+ T-cells, only three Mamu-A1*001 progressors were available. None of these RM had any detectable cytokine response in their CD8+ memory T-cell subset of BAL or PBMC (Figure 6E,F). In contrast, controllers had a total cytokine response of 1.7% in PBMC and 4.7% in BAL of CD8+ memory T-cells (Figure 6E,F) and approximately half of the cytokine secreting cells in both BAL and PBMC were polyfunctional (Data not shown).

### Controllers effectively suppress viral RNA load in blood and mucosal tissues

The highly effective reduction of systemic viral replication together with the strong virus-specific mucosal immune response, detected by tetramer staining and ICS, raised the question about the viral load in mucosal tissue. Therefore, total RNA and genomic DNA (gDNA) were isolated from BAL cells and colonic and duodenal biopsies. Viral RNA load and proviral copies were quantified by real-time PCR.

**Figure 7:**
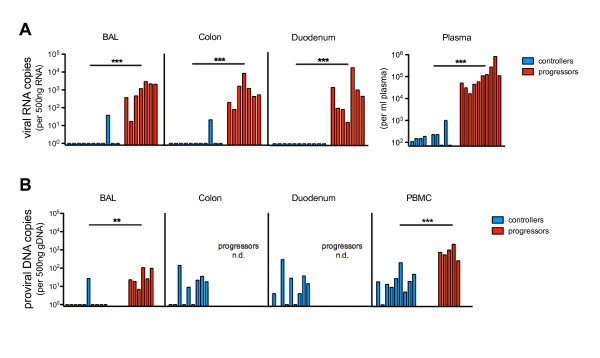
**Viral RNA and proviral load in blood and mucosal tissue of controllers and progressors**. (A) Viral RNA copies were determined per 500 ng total RNA of BAL cells, duodenal and colonic biopsies from controllers and progressors and shown along with the respective RNA viral load per ml plasma. (B) Proviral DNA copies per 500 ng genomic DNA were determined in BAL cells, PBMC, colonic and duodenal biopsies of controllers and in PBMC and BAL cells of progressors. *P*-values were calculated with the Mann-Whitney's *U*-test (**P < 0.01 and ***P < 0.001).

Surprisingly, no viral RNA was detected in BAL and intestine of controllers with the exception of one animal (12536). This animal had 37 viral copies in BAL and 20 in colon per 500 ng total RNA (Figure 7A), and the highest systemic viral load among the controllers at the respective time point (1 × 10^3 ^viral RNA copies/ml plasma). In contrast, the progressors had a significantly higher viral load not only in plasma but also in all mucosal compartments ranging from 15 to 1.5 × 10^4 ^copies per 500 ng total RNA. When taking data from controllers and progressors into account, we observed a highly significant correlation between the viral load in plasma and each mucosal compartment investigated (*P *< 0.0001).

The proviral copies in PBMC and in both intestinal sites from controllers were similar and ranged from undetectable to 3 × 10^2 ^copies per 500 ng gDNA (Figure 7B). In BAL cells from only one controller (12536), we detected 27 proviral copies per 500 ng gDNA, whereas all others were below the detection limit. Unfortunately, no gut samples of progressors were available to determine proviral load, but in BAL and PBMC we observed significantly higher proviral copy numbers than in controllers. In progressors the proviral load in BAL (7 to 1 × 10^2 ^copies) was significantly lower than in their PBMC (2.6 × 10^2 ^to 2.1 × 10^3 ^copies) (*P *= 0.0079).

## Discussion

Various studies have demonstrated a correlation between peripheral CD8+ T-cell responses and suppression of viral replication in HIV-infected humans [2,34,36] and SIV-infected RM [37,38]. However, in this context little is known about the role of the mucosal immune system. To our knowledge, this is the first comparative study with a large cohort of SIV-infected RM of Indian origin effectively controlling viral replication, which examines the immunological and virological status of different mucosal tissues *ex vivo*.

Here, we demonstrated that controllers in blood and mucosal sites exhibit (i) an effective control of viral replication (ii) have almost normal levels of CD4+ T-cells and high frequencies of Gag-specific CD8+ T-cells as well as a lower immune activation (iii) and a robust polyfunctional CD8+ T-cell response.

Mucosal tissues are major sites of viral replication [22,24,25], but interestingly, our controllers were able to reduce viral RNA load not only in blood but also in all mucosal tissues investigated. Since during the acute phase of HIV infection a reservoir of latently infected resting CD4+ T-cells is established, with a mean half-life of about 3.5 years [39], it follows that proviral DNA would be detected not only in progressors but also in the majority of samples from controllers. However, almost all BAL samples from controllers were negative for SIV provirus and in progressors the proviral load was significantly lower than in their PBMC which might be explained by the higher cell turnover on the lung surface.

All studies with pathogenic SIV infection in RM investigating mucosal tissues during peak viremia reported a dramatic loss of CD4+ T-cells in the gut [11,12,22,26,40], the female genital tract [23] and BAL [41]. To date, a repopulation of mucosal CD4+ T-cells has only been demonstrated in SIV-infected Chinese RM, which control viral replication and moreover analyzing just one mucosal site [40,41]. However, the course of disease is attenuated in these monkeys compared to RM of Indian origin used in this study.

When analyzing blood and three different mucosal sites from our controllers of Indian origin, we found in blood, BAL, duodenum and colon almost normal CD4+ T-cell levels, which significantly exceed those of progressors. We demonstrated that controllers naturally and effectively suppress viral replication in blood and mucosal organs, which is accompanied by a repopulation of CD4+ T-cells in all mucosal tissues albeit to a varying degree. Almost normal CD4+ T-cell levels combined with low proportions of CD4+CCR5+ T-cells in both gut sites of controllers argues for a repopulation of mainly CD4+CCR5- T-cells. The reduction of the primary viral target cells in the intestine, the largest mucosal organ, may significantly contribute to long-term control of viral replication.

By using tetramer technology we demonstrated a higher systemic, and especially mucosal, Gag-specific cellular immune response in controllers than in progressors. We confirmed with the longitudinal analyses of controllers for up to three years, that the levels of these virus-specific T-cells are relatively stable in blood and all three mucosal tissues, combined with persistently high levels of CD4+ T-cells and low viral loads.

One former controller (12536) displayed a slowly increasing plasma and mucosal viral load (Data not shown) over two years, accompanied by a severe decrease of Gag-specific T-cells, but surprisingly stable levels of CD4+ T-cells in blood and all mucosal tissues. This points to an as yet undefined mechanism, that in former controllers blood and mucosal CD4+ T-cells can be preserved for an unknown period of time despite increasing viral replication obviously decelerating the progression to AIDS like disease, as this animal remains healthy to date (5 years post infection).

During HIV infection, a chronic immune activation correlates with high viral load, systemic CD4+ T-cell depletion and a faster disease progression [28,31,32,42]. Our results are in line with these findings, as we observed a lower HLA-DR expression on CD4+ and CD8+ T-cells in blood and mucosal tissues of controllers compared to progressors.

However, when considering only the controller cohort in detail, we observed that the relationship between immune activation, virus-specific T-cells and CD4+ T-cell levels is not only restricted to individuals in general but also to single organs in particular.

In the blood and duodenum of controllers, we found rather lower levels of Gag-specific T-cells and significantly decreased proportions of CD4+ T-cells with a significantly higher expression of HLA-DR. The opposite pattern was found in BAL and colon, where the CD4+ T-cells and their HLA-DR expression did not differ from uninfected RM and the mean levels of virus-specific T-cells were higher than in blood and duodenum. These results clearly suggest a direct association between virus-specific immune response, CD4+ T-cell levels and their activation level within single organs.

In contrast to PBMC, functional characterization of mucosal cells is generally more complex and time-consuming. In RM, it is hardly feasible to collect as many intestinal biopsies as in humans, thus ending up with much lower cell yield and almost precluding a functional characterization by ELISpot or ICS. Moreover, to obtain intestinal cells, the biopsies have to be digested enzymatically, which may influence cytokine secretion. Therefore, we used easily accessible BAL cells for a functional characterization of the mucosal immune system.

Our data demonstrated, that the total CD8+ cytokine response was significantly higher in PBMC and BAL cells of controllers than in progressors, when stimulated with the Gag-peptides. Of note, the frequencies of polyfunctional Gag-specific CD8+ T-cells in BAL were significantly higher than in progressors, this did not, however, apply for blood. When comparing mucosal and systemic responses, the different ratios between naïve and memory cells must be considered because mucosal tissues exhibit significantly more memory T-cells than PBMC [43] and virus-specific cytokine secretion is restricted to memory cells [44]. Therefore, we excluded naïve cells from analyses and displayed the cytokine secreting cells as a proportion of memory cells. Both total and polyfunctional CD8+ BAL responses in controllers against the Gag-peptide pool and Gag-CM9 significantly exceeded their respective responses in blood. This suggests that a robust CD8+ virus-specific polyfunctional mucosal immune response is even more important than a peripheral one to control viral replication.

Only a few studies investigated mucosal immune responses in controller individuals, but detailed mucosal immune analyses of intestinal lymphocytes from well-defined cohorts including HIV controlling individuals reported recently a strong CD8+ and CD4+ dependent rectal mucosal immune response associated with viral suppression [45-47]. In contrast to these findings, we did not observe a difference between controllers and progressors regarding their virus-specific CD4+ response. However, the cytokine secretion in their studies was related to the total amount of CD4+ or CD8+ T-cells and the different ratio between naïve and memory T-cells in blood and gut was not considered.

In addition, not only the virus-specific stimulation, but also the polyclonal stimulation of PBMC and BAL cells with SEB provided important information. Comparing the functionality of peripheral T-cells from controllers, progressors and uninfected RM after SEB stimulation displayed hardly any significant differences between these animal cohorts. In contrast, the cytokine responses of CD4+ and CD8+ memory T-cells in BAL of controllers were slightly reduced compared to uninfected animals but not to the same extent as in progressors. These results suggest an irreversible damage of the mucosal immune system that probably occurred during peak viremia and cannot be recovered completely, even in controllers displaying a robust suppression of viral replication. Of note, the frequencies of polyfunctional CD8+ cells as well as the total cytokine response of CD4+ and CD8+ memory T-cells were still significantly higher in controllers than in progressors. Possibly the stimulation of BAL cells with SEB can be a comparatively easy method providing prognostic information about the functional status of the mucosal immune system in the lung of HIV/SIV-infected individuals.

## Conclusion

Our study demonstrated that a functional virus-specific mucosal immune response significantly contributes to an efficient overall reduction of viral replication and is associated with a repopulation of CD4+ T-cells in different mucosal organs. We conclude that, inducing a strong mucosal immune response during vaccination might lead to a later controller status and therefore could be a stepping-stone to developing a protective vaccine with sterilizing immunity.

## Methods

### Animals, blood and tissue sampling

For this study 45 adult colony-bred rhesus monkeys of Indian origin comprising 15 naïve and 30 experimentally infected with SIVmac239 or SIVmac251, between 4 and 12 years old were used. The animals were housed at the German Primate Center under standard conditions according to the German Animal Welfare Act, which complies with the European Union guidelines on the use of non-human primates for biomedical research. MHC alleles were typed using allele- or group specific primers as described [48].

To collect bronchoalveolar lavage, intestinal biopsies and blood the animals were anesthetized with a mixture of 5 mg ketaminhydrochloride, 1 mg xylazinhydrochloride and 0.01 mg atropine sulfate per kg body weight. Mucosal cells were isolated as previously described [43]. PBMC for IFN-γ ELISpot, intracellular cytokine staining and proviral load were isolated from peripheral blood using Ficoll-hypaque density centrifugation. The different assays were performed at various time points, but within each analysis paired samples from different compartments of each animal were taken and analyzed. All SIV-infected animals were derived from different vaccine experiments, but there was no correlation between previous vaccination received and the ensuing controller or progressor status. Moreover, in both animal cohorts, controllers and progressors, there was no difference between the viral loads of vaccinated and unvaccinated RM over the whole period of infection.

### ELISpot

IFN-γ ELISpot assay was performed using SIV-Gag (EVA7066, NIBSC, UK); Env2 (6583-6637, NIH), Nef (EVA777, NIBSC) and Tat (EVA7069, NIBSC) peptides as described previously [49]. The IFN-γ positive cells were counted using a Bioreader^®^-3000 (Bio-Sys GmbH, Karben, Germany). Individual values were obtained by peptide stimulation minus medium control and considered positive when exceeding 100 spot forming cells (SFC) per million PBMC.

### ELISA

To detect antibodies against SIV, a standard ELISA [50] was performed on plates coated with 30 ng per well of recombinant SIV-p27 (EVA643, NIBSC) or recombinant SIV-gp130 (EVA670, NIBSC). Anti-SIV-IgG antibodies were determined by end-point-dilution. The titers were expressed as the reciprocal of the highest dilution yielding optical densities twice above the autologous preinfection or preimmunization values.

### Monoclonal antibodies and flow cytometric surface staining

Different lymphocyte populations were investigated by polychromatic flow cytometry. For surface staining, 50 μl of whole blood and 0.4-1 × 10^6 ^mucosal cells were analyzed using the following monoclonal antibodies from BD Bioscience (Heidelberg, Germany): anti-CD3 Alexa700 (clone SP34-2), anti-CD4 PacificBlue (clone L200) or CD4-Alexa405 (clone L200), anti-CD8 AmCyan (clone SK1), CCR5-APC (clone 3A9), HLA-DR-APC-Cy7 (clone L234). For tetramer analysis the CM9-tetramer-PE (Beckman Coulter, Krefeld, Germany) was used. Twelve-parameter flow cytometric analysis was performed using a BD LSRII flow cytometer (BD Biosciences) and the list-mode data files were analyzed using FlowJo Version 8.7 (Tree Star).

### Intracellular cytokine staining

Freshly isolated PBMC or BAL cells were resuspended in RPMI 1640 medium (PAN Biotech, Aidenbach, Germany) supplemented with 10% heat inactivated FCS and 1 μg/ml anti-CD28 costimulatory antibody. 0.7-1.5 × 10^6 ^cells were stimulated with synthetic 15 mer Gag peptide pool (EVA7066, NIBSC, final concentration 2 μg/ml/peptide), with the single CM9-peptide (Gag_181-189_, CTPYDINQM, final concentration 2 μg/ml) or with *Staphylococcus *enterotoxin B (SEB; final concentration 1 μg/ml) respectively. Additionally, two negative controls were performed, one containing a peptide pool from human hepatitis C virus and one anti-CD28 only. The cultures were incubated for 1 h at 37°C in a 5% CO2 incubator, followed by a 5 h incubation in the presence of Brefeldin A (10 μg/ml; Sigma-Aldrich, Taufkirchen, Germany). After washing the surface staining was performed with anti-CD3 Alexa700 (clone SP34-2, BD Bioscience), anti-CD4 PerCPCy5.5 (clone L200, BD Bioscience), anti-CD8 PacificOrange (clone 3B5, CALTAG, Buckingham, UK) and anti-CD45-RA-ECD (clone 2H4, Beckman Coulter) antibodies for 30 min at room temperature and then fixed with 4% formaldehyde in PBS for 10 min at 37°C. Cells were stored overnight in 400 μl PBS-buffer at 4°C and stained on the following day with anti-IL-2 FITC (clone MQ1-17H12, BD Bioscience), anti-TNF-α PE (clone MAb11, BD Bioscience), and anti-IFN-γ APC (clone B27, BD Bioscience) PBS-buffer containing 0.5% Saponin for 45 min at 4°C. After a final wash cells were resuspended in PBS and analyzed by flow cytometry (BD LSRII flow cytometer). Background correction was done by using the anti-CD28 negative control. Boolean-gating identified single positive cells (secreting only one cytokine) and polyfunctional (producing two or three cytokines) CD45RA- memory T-cells. A SIV-specific positive response was defined by reaching at least twice the height of the HCV-response.

### SIV viral load

Viral RNA was isolated from frozen plasma samples following the MagAttract Virus Mini M48 protocol (Qiagen, Hilden, Germany) and total RNA from intestinal biopsies and BAL-cells was isolated using Qiagen RNeasy Plus Mini Kit in accordance with the manufacturer's protocol. Viral RNA copies were quantified in purified SIV RNA from plasma or in 500 ng total RNA from tissue using TaqMan-based real-time PCR on an ABI-Prism 7500 sequence detection system (Applied Biosystems) as described [51]. As an internal standard, the amount and quality of total RNA from mucosal tissues was compared to the GAPDH-house keeping gene in parallel.

For detecting SIV, proviral load genomic DNA was isolated from PBMC, BAL cells and intestinal biopsies using the QiaAmp DNA Mini Kit (Qiagen) according to the manufacturer's instructions including a RNA digestion. SIV proviral copies were quantified using a total amount of 500 ng of isolated DNA, the Gene Expression Master Mix (Applied Biosystems) TaqMan-based real-time PCR on an ABI-Prism 7500 sequence detection system (Applied Biosystems) as described before [51].

### Statistical analysis

Comparison between two animal groups or different tissues was performed using two-tailed Mann-Whitney's *U*-test in GraphPad Prism software, version 5 (GraphPad Software, San Diego, USA). Correlation analyses between variables were performed by using the Spearman correlation (GraphPad Prism).

For correlations including the plasma viral load, we used the individual viral RNA copy numbers per ml plasma of each animal at the respective point in time when the assay was performed.

## Competing interests

The authors declare that they have no competing interests.

## Authors' contributions

TS performed flow cytometric surface analysis of blood and mucosal cells as well as the intracellular cytokine staining, quantified SIV RNA load in mucosal tissue and wrote the manuscript. RS helped with all flow cytometric analyses and wrote the manuscript. US quantified the SIV RNA load in plasma, analyzed the MHC genotypes and reviewed the manuscript. WI performed the SIV proviral load analyses. CSH carried out ELISA and ELIspot experiments and reviewed the manuscript. TS, RS and CSH designed and coordinated the study and edited the manuscript. All authors read and approved the final manuscript.

## Supplementary Material

Additional file 1 **Table S1. MHC class I genotypes**. MHC class I background of SIV-infected rhesus monkeys used in this study. MHC alleles were typed using allele- or group specific primers as described [48] and genotypes associated with slow disease progression are in bold letters. Of one MamuA1*001 negative progressor (12539) no DNA-samples for further typing were available, so that additional data are not presented. Click here for file
