# Supplementary material for: Strong mucosal immune responses in SIV infected macaques contribute to viral control and preserved CD4+ T-cell levels in blood and mucosal tissues
Source: Retrovirology. 2011 Apr 11;8:24. doi: 10.1186/1742-4690-8-24 (PMC3096904; doi:10.1186/1742-4690-8-24)
Supplement: Additional file 1 — Table S1. MHC class I genotypes. MHC class I background of SIV-infected rhesus monkeys used in this study. MHC alleles were typed using allele- or group specific primers as described [48] and genotypes associated with slow disease progression are in bold letters. Of one MamuA1*001 negative progressor (12539) no DNA-samples for further typing were available, so that additional data are not presented. [file 1742-4690-8-24-S1.DOC]

**Table S1. MHC class I genotypes**

|  | MHC class I A* | MHC class I B* |
| --- | --- | --- |
| **Controllers** |  |  |
| 2139 | ***A1*001****, A1*004, A1*008, A2*05, A3* | ***B*017****, B*021, B*028, B*029, B*045, B*061* |
| 2151 | ***A1*001****, A1*004, A1*008, A2*05, A3* | ***B*017****, B*021, B*028, B*029, B*045, B*061* |
| 2153 | ***A1*001****, A1*004, A1*008, A2*05, A3* | ***B*017****, B*021, B*028, B*029, B*045, B*061* |
| 2155 | ***A1*001****, A1*004, A1*008, A2*05, A3* | ***B*017****, B*021, B*028, B*029, B*045, B*061* |
| 2172 | *A1*004, A1*008, A4*14* | *B*012, B*021,B*022, B*028, B*030, B*045, B*046, B*049, B*057* |
| 2191 | ***A1*001****, A1*011* | *B*038,B*046,* ***B*047:01*** |
| 8644 | ***A1*001****, A1*008, A2*05, A3* | *B*038,* ***B*047:01*** |
| 9045 | ***A1*001,*** *A1*008, A2*05, A4*14* | *B*057, B*046* |
| 9794 | *A1*002, A1*004, A3*013, A4*14* | ***B*017****, B*029, B*041, B*048, B*061* |
| 12533 | ***A1*001****, A2*05, A1*004, A4*14* | *B*001, B*007, B*043, B*030, B*057, B*068* |
| 12535 | *A1*004, A1*008, A2*05, A4*14* | *B*001, B*007,* ***B*017****, B*029, B*030, B*057, B*075* |
| 12536 | ***A1*001*** | *B*057, B*048, B*075* |
| 12671 | ***A1*001****, A*004, A2*05, A4*14* | *B*006,* ***B*008****, B*019, B*024, B*046, B*057, B*082* |
| 12672 | *A1*008, A4*14, A3* | *B*012, B*022, B*048, B*065, B*075* |
| **Progressors** |  |  |
| 2118 | ***A1*001****, A1*002, A2*05, A3* | *B*001, B*007, B*012, B*022, B*030, B*049, B*057* |
| 2141 | *A1*004, A1*008, A4*14, A3* | *B*021, B*028, B*041, B*045, B*048, B*064* |
| 2188 | ***A1*001****, A2*05, A3* | ***B*047:01****, B*024* |
| 12537 | *A1*004, A2*05, A4*14* | *B*001, B*007, B*030, B*046, B*057* |
| 2168 | *A1*004, A1*008, A4*14 , A3* | *B*012, B*022, B*030****, B*047:01****, B*038, B*046, B*057* |
| 10425 | *A1*004, A2*05, A4*14* | *B*041, B*046, B*048, B*064, B*065, B*069* |
| 2192 | *A1*004, A1*008, A4*14* | *B*012, B*021,B*022, B*028, B*030, B*045, B*046, B*049, B*057* |
| 12531 | *A1*004, A1*011, A2*05, A4*14* | *B*065, B*001, B*007, B*012, B*030, B*057, B*049, B*068* |
| 12538 | ***A1*001,*** *A2*05* | *B*001, B*007, B*030, B*057* |
| 11139 | ***A1*001****, A2*05* | *B*012, B*030, B*041, B*046, B*048, B*057, B*064, B*065* |
| 13251 | ***A1*001,*** *A2*05* | *B*001, B*007, B*030, B*057* |
| 13258 | *A1*004, A4*14* | ***B*008****, B*046, B*057* |
| 13250 | ***A1*001****, A2*05, A3, A4*14* | *B*001, B*007, B*030, B*057* |
| 13257 | *A1*004, A1*008, A2*05, A4*14* | *B*012,* ***B*017****, B*029* |
| 13260 | *A2*05, A4*14* | *B*046, B*047, B*065, B*057* |
